# Comparison of Prognostic Genomic Predictors in Colorectal Cancer

**DOI:** 10.1371/journal.pone.0060778

**Published:** 2013-04-23

**Authors:** Yun-Yong Park, Sung Sook Lee, Jae Yun Lim, Sang Cheol Kim, Sang Bae Kim, Bo Hwa Sohn, In-Sun Chu, Sang Cheul Oh, Eun Sung Park, Woojin Jeong, Sung Soo Kim, Scott Kopetz, Ju-Seog Lee

**Affiliations:** 1 Departments of Systems Biology, Division of Cancer Medicine, The University of Texas MD Anderson Cancer Center, Houston, Texas, United States of America; 2 Korean Bioinformation Center, Korea Research Institute of Bioscience and Biotechnology, Daejeon, Korea; 3 Institute for Medical Convergence, Yonsei University College of Medicine, Seoul, Korea; 4 Department of Life Science, Division of Life and Pharmaceutical Sciences, Center for Cell Signaling and Drug Discovery Research, Ewha Womans University, Seoul, Korea; 5 Gastrointestinal Medical Oncology, Division of Cancer Medicine, The University of Texas MD Anderson Cancer Center, Houston, Texas, United States of America; 6 Department of Internal Medicine, Yonsei University College of Medicine, Seoul, Korea; 7 The University of Texas Graduate School of Biomedical Sciences, Houston, Texas, United States of America; 8 Division of Hemato-Oncology, Department of Internal Medicine, Korea University Medical Center, Korea University College of Medicine, Seoul, Korea; 9 Department of Biochemistry and Molecular Biology, Medical Research Center for Bioreaction to Reactive Oxygen Species and Biomedical Science Institute, School of Medicine, Seoul, Korea; Duke-National University of Singapore Graduate Medical School, Singapore

## Abstract

**Background:**

Although several prognostic genomic predictors have been identified from independent studies, it remains unclear whether these predictors are actually concordant with respect to their predictions for individual patients and which predictor performs best. We compared five prognostic genomic predictors, the V7RHS, the ColoGuideEx, the Meta163, the OncoDX, and the MDA114, in terms of predicting disease-free survival in two independent cohorts of patients with colorectal cancer.

**Study Design:**

Using original classification algorithms, we tested the predictions of five genomic predictors for disease-free survival in two cohorts of patients with colorectal cancer (n = 229 and n = 168) and evaluated concordance of predictors in predicting outcomes for individual patients.

**Results:**

We found that only two predictors, OncoDX and MDA114, demonstrated robust performance in identifying patients with poor prognosis in 2 independent cohorts. These two predictors also had modest but significant concordance of predicted outcome (r>0.3, *P*<0.001 in both cohorts).

**Conclusions:**

Further validation of developed genomic predictors is necessary. Despite the limited number of genes shared by OncoDX and MDA114, individual-patient outcomes predicted by these two predictors were significantly concordant.

## Introduction

Colorectal cancer is the second-leading cause of death from cancer in the United States, and about 40% of new cases are diagnosed while the cancer is in the early or localized stage [1]. Because accurate prognosis is essential for selecting the most effective treatment, considerable effort has been devoted to establishing a colorectal cancer stratification (or staging) model for, using clinical information and pathological criteria. Although clinicopathological staging systems such as the Dukes system and the American Joint Committee on Cancer (AJCC) system have been the gold standards as prognostic indicators [2]–[4], developing improved prognostic tools is important because the clinical predictors used currently provide only broad categorization of risk and fail to identify biological characteristics important for matching patients with specific therapies.

With the recent advent of microarray technology, risk assessment for colorectal cancer has been improved by using gene expression profiling. Researchers at the Ludwig Institute for Cancer Research and the H. Lee Moffitt Cancer Center identified a gene expression signature that can predict distant metastasis of colorectal cancer [5]. A similar genomic prognostic predictor was developed at The University of Texas MD Anderson Cancer Center [6]. In a different study, seven genes were identified as a minimum prognostic gene set, and risk scores for recurrence were subsequently developed later [7]. Other prognostic genomic predictors include Oncotype DX (OncoDX), ColoPrint, ColDx, and ColoGuideEx [8]–[11].

Although genes in each prognostic indicator overlap minimally with those in the other predictors, whether these genetic signatures identify the same population of patients is unclear [12]. Additionally, the predictive accuracies of the indicators have never been directly compared in the same cohort of patients with colorectal cancer. Thus, the question of whether these predictors are concordant in predicting outcomes for individual patients and the question of which predictor performs best have not been resolved previously. In this study, we used various statistical approaches to determine the concordance of several genomic predictos in predicting clinical outcomes of individual patients in two independent cohorts.

## Materials and Methods

### Prognostic genomic predictors

**Figure 1:**
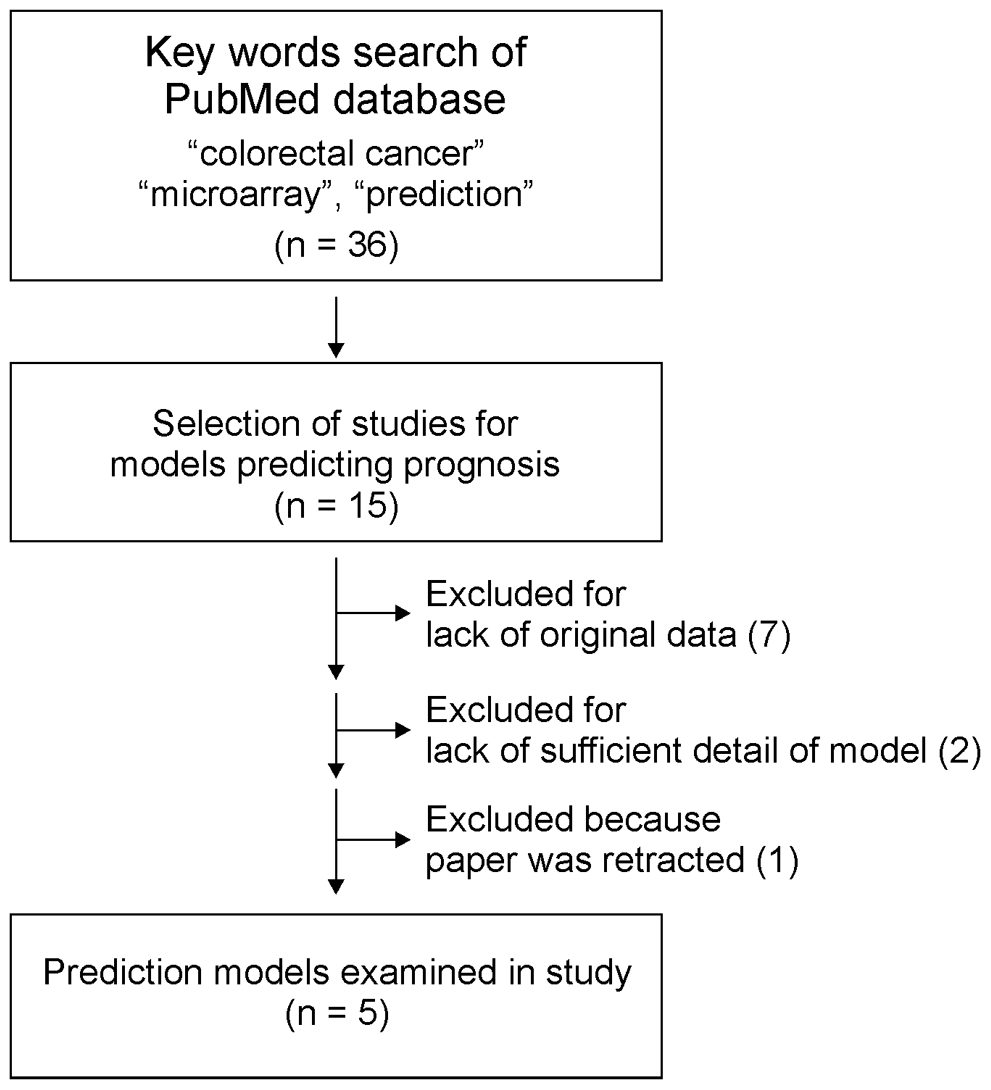
CONSORT flow diagram for selection of prediction models.

Using the search terms “colorectal cancer”, “microarray”, and “prediction”, we searched the PubMed database for previously published studies on prognostic genomic predictors (**Figure 1**). This search led us to 36 microarray-based studies on colorectal cancer. After looking at articles referenced in these studies, we identified 15 studies that had carried out microarray or reverse transcriptase-polymerase chain reaction experiments to develop gene expression-based prognostic predictors [5]–[11], [13]–[20]. In those studies, detailed descriptions of the prediction models and their associated original gene expression data were provided for a total of six genomic predictors; we selected these six predictors for further analysis (the remaining nine studies were excluded because they lacked a full description of the prediction model or primary data). Of the six selected studies, we excluded the Dukes 50-gene predictor because the original paper was retracted [18], [21].

The 5 prognostic genomic predictors we examined were the (i) Veridex 7-gene relapse hazard score (V7RHS) developed by Jiang et al. [7], (ii) metastasis-associated 163-gene expression signature (Meta163) developed by Jorissen et al. [5], (iii) 7-gene Oncotype DX recurrence score (OncoDX) developed by Genomic Health [8], (iv) 114-gene MD Anderson Cancer Center prognostic predictor (MDA114) developed by Oh et al. [6], and (v) 13-gene ColoGuideEx prognostic predictor developed by Agesen et al. [11].

### Patients and genomic data

Data with the Gene Expression Omnibus (GEO) accession number GSE14333 had been generated from fresh-frozen tumor specimens that had been retrieved from the tissue banks of the Royal Melbourne Hospital, Western Hospital, and Peter MacCallum Cancer Center in Australia and of the H. Lee Moffitt Cancer Center in the United States (Australian-US [AUS] cohort, n = 229) (**Table 1**) [5]. Of the 229 patients in the AUS cohort, 87 had received standard adjuvant chemotherapy (either single-treatment 5-fluorouracil/capecitabine or a combination of 5-fluorouracil and oxaliplatin). The remaining 142 patients had not received chemotherapy. Disease-free survival (DFS) was defined in a previous study as the time from surgery to the first confirmed relapse; data had been censored when a patient died or was alive without recurrence at last contact [5]. Data with accession numbers GSE17538 and GSE37892 had been generated from fresh-frozen tumor specimens of patients at the Vanderbilt Medical Center and Institut National de la Santé et de la Recherche Médicale (INSERM), respectively. The two pooled data sets were correspond to the VI cohort (n = 168) [19], [22].

**Table 1:** Clinical and pathological characteristics of patients with colorectal cancer.

Characteristics	AUS cohort (N = 229)	VI cohort (N = 168)
**Sex**		
Male	123	90
Female	106	78
**Age**		
Median	67	68
Range	26–92	22–97
**Site**		
Colon	199	168
Rectum	30	0
**AJCC Stage**		
** ** **I**	44	4
** ** **II**	94	88
** ** **III**	91	76
**Median Follow-up**	47.5 month	50.9 month
**Chemotherapy**		
Yes	87	NA*
No	142	NA
**Radiation therapy**		
Yes	22	NA
No	207	NA

* NA, Not Available

Gene expression data had been generated by using the Affymetrix U133 version 2.0 platform. Raw data were downloaded from the GEO database and normalized using a robust multiarray averaging method [23].

### Stratification of patients according to prognostic genomic predictors

For patient stratification according to the V7RHS, the log2-transformed expression level of seven genes (*YWHAH*, *CAPG*, *KLF5*, *EPM2A*, *LAT*, *LILRB3*, and *RCC1* [also known as *CHC1*]) in the AUS patients were normalized by subtracting the average expression values of three housekeeping control genes (*ACTB*, *HMBS*, and *RPL13A*) to generate ΔCt values. These 3 genes had been selected as controls in the original study [7]. The V7RHS for each patient was derived by summation of the multiplication of the expression (ΔCt) values of a gene with its corresponding coefficients generated from the published regression model (−3.156 for *YWHAH*, −2.842 for *CAPG*, +3.002 for *KLF5*, −2.835 for *EPM2A*, −3.249 for *LAT*, −3.215 for *LILRB3*, and −3.036 for *RCC1*). Patients with a V7RHS >0 were classified as high risk, and those with a V7RHS <0 were classified as low risk as described previously [7].

The MA163 predictor was developed with gene expression data from a subset of the AUS cohort, so we followed the same stratification strategy as that used in a previous study [5]. Briefly, expression data of 163 gene features in the training set (Dukes stages A and D) were combined to form a classifier according to the nearest centroid algorithm. The nearest centroid estimates the probability that a particular sample belongs to stage A or stage D. The trained predictor was directly applied to the test set (Dukes stages B and C) to identify stage A-like and stage D-like patients.

A microarray-based approximation of OncoDX was calculated by using recurrence score algorithms modified for data from microarray experiments [8]. First, 12 genes (7 recurrence genes and 5 reference genes) were identified by using gene symbols. When multiple probes in the Affymetrix platform represented the same gene, the gene probes with the highest variance in the gene expression pattern were selected over others. Second, the expression levels of the seven recurrence genes (*BGN*, *MYC*, *FAP*, *GADD45B*, *INHBA*, *MK167*, and *MYBL2*) were then normalized by dividing the mean expression levels of the five reference genes (*UBB*, *ATP5E*, *PGK1*, *GPX1*, and *VDAC2*). Third, for the lowest level of gene expression to equal zero as in the previous study [8], the normalized expression level of each gene was subtracted by the minimum expression values across all seven recurrence genes. Reference normalized expression measurements from microarray experiments range from 0 to 6.2 on a log2-scale. Fourth, the mean expression level of each group—cell cycle group (*MYBL2*, *MKI67*, and *MYC*), stromal group (*BGN*, *FAP*, *INHBA*), and *GADD45B* alone—represented group scores as described in the original algorithm. The unscaled recurrence score (RSu) was calculated with the use of pre-determined coefficients: 0.1263× stromal group score–0.3158× cell cycle group score +0.3406× GADD45B score. Fifth, the recurrence score was rescaled by multiplying it by 44.16 after adding 0.3 to each RSu according to the original algorithm, and then subtracting the minimum values of scores across all patients. Subtraction of the recurrence score (which made the lowest score equal to 0) was necessary because the lowest recurrence score was defined as 0 by the original algorithm. Rescaled recurrence scores ranged from 0 to 88.5. Patients were then stratified according to the original cut-off for OncoDX by risk group: low risk, <30; intermediate risk, 30 to 40; high risk >40. When patients were stratified into 2 groups, patients with a risk score of 30 or higher were considered at high risk.

For patient stratification according to the MDA114 predictor, gene expression data from the original training data set (GSE17538) were used to train a predictor, and those from patients in the AUS cohort were used as a test data set as described previously [6]. Briefly, a compound covariate predictor algorithm was applied first to the training data set for training of the predictor and then later to patients in the AUS data set to stratify patients into two recurrence risk groups, high and low.

With the ColoGuideEx predictor, patients were stratified according to the number of genes exceeding the 80th and 20th percentile levels of each gene in the ColoGuideEx signature [11]. High-risk genes (genes whose expression is high in patients with poor prognosis) are *AZGP1*, *BNIP3*, *DSC3*, *ENPP3*, *EPHA7*, *KLK6*, *SEMA3A*, and *SESN1*. Low-risk genes (genes whose expression is low in patients with poor prognosis) are *CXCL10*, *CXCL13*, *MMP3*, *PIGR*, and *TUBA1B*. For each patient, the number of high-risk genes whose expression level was above the 80th percentile and the number of low-risk genes whose expression level was below the 20th percentile were counted. The numbers of genes ranged from 0 to 8. Patients with more than 5 genes were considered at high risk.

### Statistical analysis

Before we applied the prognostic classification algorithms, gene expression data used as training and test data sets were normalized by centralizing the gene expression level across the tissues. The BRB-ArrayTools was used for statistical analysis of gene expression data [24]. We estimated patient prognoses by using Kaplan-Meier plots and the log-rank test. We then used multivariate Cox proportional hazards regression analysis to evaluate independent prognostic factors associated with survival; we used gene signature, tumor stage, and pathological characteristics as covariates [25]. A *P* value less than 0.05 indicated statistical significance, and all statistical tests were two-tailed. To assess the strength of the correlation between outcomes predicted by the different predictors, we applied Cramer's V statistics and two-way contingency-table analyses. All statistical analyses were conducted in the R language environment [26].

Receiver-operating characteristic (ROC) curve analyses were carried out to estimate the discriminatory power of the prognostic genomic predictors. Area under the curve (AUC) ranged from 0.5 (for a noninformative predictive marker) to 1 (for a perfect predictive marker). A bootstrap method (1000 re-sampling) was used to calculate the 95% confidence interval (CI).

## Results

### Robustness of prognostic genomic predictors

The number of unique genes in each of the five prognostic genomic predictors ranged from 7 to 121 (**Table S1**). Only a few genes appear on more than one in the five gene lists. MA163 and MDA114 have the largest numbers of genes, but they share only seven (**Table S2**).

**Figure 2:**
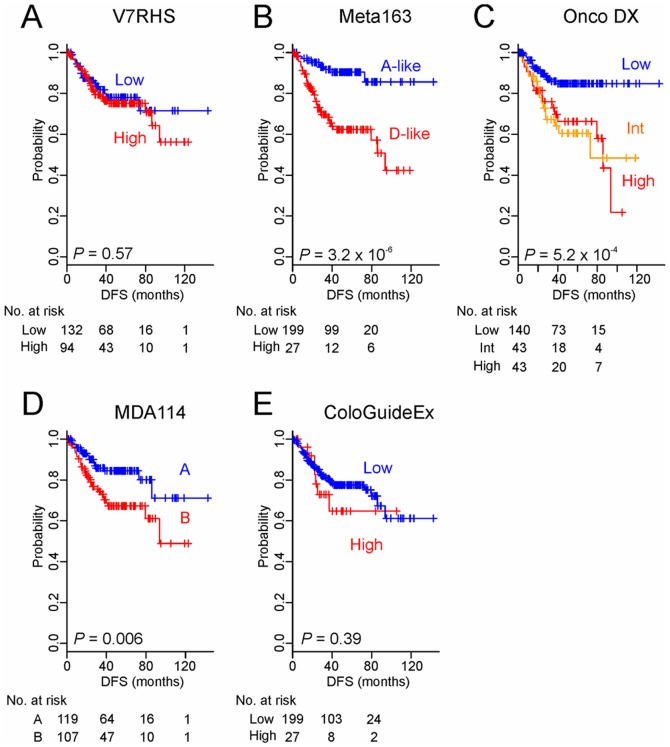
Kaplan–Meier survival plots of the DFS rates of AUS patients stratified by risk level according to the five genomic predictors (A to E). DFS data were not available from three patients. *P* values are based on the log-rank test. Int, intermediate.

By applying the original prediction algorithms and cut-off values developed in previous studies, we first stratified patients in the AUS cohort according to the risk level predicted by the five genomic predictors. Three of the predictors showed significant association with prognosis; Kaplan-Meier plots and log-rank tests showed significant differences between the DFS rates of patients with a high-risk and the DFS rates of those with a low-risk (**Figure 2**). Split ratios of patients in the predictors varied from 11.8% and 88.2% (ColoGuideEx) to 51.5% and 48.5% (Meta163) (**Table S3**).

**Figure 3:**
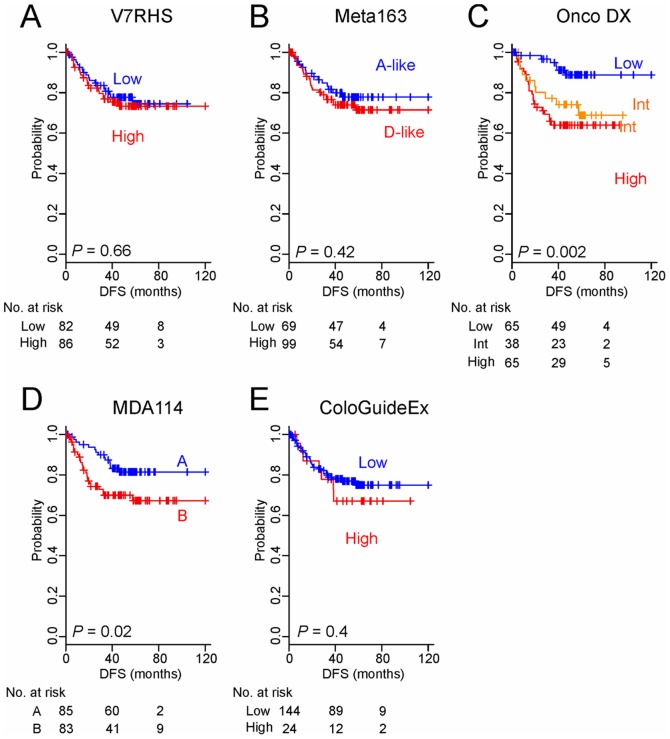
Kaplan–Meier survival plots of the DFS rates of VI patients stratified by risk level according to the five genomic predictors (A to E). *P* values are based on the log-rank test. Int, intermediate.

To further test the reproducibility and robustness of the predictions made on the basis of five predictors, we stratified patients in the VI cohort (n = 168). As with the AUS cohort, OncoDX and MDA114 showed significant association with prognosis (**Figure 3**). Thus, predictions for colorectal cancer patients made on the basis of only two of the five indicators were reproducible for the two cohorts.

### Concordance between predictors

We next evaluated concordance between the predicted outcomes by comparing the membership of patients predicted for each risk level. Predicted outcomes varied between the predictors. For example, of the 86 patients predicted by OncoDX to have a high or intermediate risk of recurrence, 63 and 60 were classified by Meta163 and MDA114, respectively, as having poor prognosis (**Table S3 and** **Figure 1**). Likewise, of the 107 patients predicted by MDA114 to have a high risk of recurrence by MDA114, only 10 and 22 patients were predicted by ColoGuideEx and V7RHS, respectively, to have poor prognosis.

To quantify concordance between the predictors, we applied Cramer's V statistics and analyzed two-way contingency-tables for the AUS cohort (**Table 2**). The highest correlations were observed between OncoDX and MDA114 in the AUS cohort (r = 0.36 by V statistics, *P* = 1.3×10^−7^ by χ^2^ test). The correlation between OncoDX and Meta163 was high (r = 0.34 by V statistics, *P* = 6.8×10^−7^ by χ^2^ test); correlation between MDA114 and Meta163 was lower (r = 0.19 by V statistics). When Cramer's V statistics were applied to predicted outcomes in the VI cohort, only the correlation between OncoDX and MDA114 remained significant (r = 0.39 by V statistics, *P* = 7.5×10^−7^ by χ^2^ test) (**Table S4**). These results suggest that the genomic predictors had only modest concordance. It is noteworthy that only the robust predictors OncoDX and MDA114 were significantly concordant (**Table 2** **and Table S4**).

**Table 2:** Concordance of the five genomic predictors in grouping AUS patients by risk level.

Predictors	V7RHS	Meta163	Oncotype DX	MDA114	ColoGuideEx
V7RHS	1	0.08	0.33*	0.4*	0.04
Meta163	0.08	1	0.34	0.19	0.16
Oncotype DX	0.33*	0.34	1	0.36	0.11
MDA114	0.4*	0.19	0.36	1	0.07
ColoGuideEx	0.04	0.16	0.11	0.07	1

Correlation was quantified using Cramer's V statistics. * Inverse correlation

### Genomic predictors in relation to clinical variables

Next, we performed univariate Cox analysis with traditional clinical pathological parameters (gender, age, tumor location, adjuvant chemotherapy, and AJCC stage) to compare their prognostic accuracy with that of each predictor (**Table S5 and S6**). In agreement with previous analyses, only MDA114 and Onco DX had significant hazard ratios (HRs) similar to AJCC stages, for both tested cohorts. We next performed multivariate Cox analysis by individually analyzing each predictor (**Table 3**). V7RHS, ColoGuideEx, and Meta163 were not included in this analysis due to their lack of association with prognosis in univariate analyses for both cohorts. For the AUS cohort, MDA114 (HR, 2.26; 95% CI, 1.25–4.1; *P* = 0.007) and OncoDX (HR, 2.38; 95% CI, 1.32–4.27; *P* = 0.003) were independent variables for predicting DFS.

**Table 3:** Multivariate Cox proportional hazard regression analyses of DFS with clinical variables and genomic predictors.

	Oncotype DX		MDA114	
Variable	Hazard Ratio (95% CI)	*P*-value	Hazard Ratio (95% CI)	*P*-value
**Gender (male or female)**	0.99 (0.56–1.7)	0.99	1.03 (0.58–1.8)	0.89
**Age (>70 or not)**	0.8 (0.43–1.48)	0.48	0.94 (0.5–1.7)	0.85
**Location (colon or rectum)**	1.23 (0.54–2.8)	0.6	1.3 (0.57–2.9)	0.52
**Chemotherapy (yes or no)**	0.8 (0.42–1.53)	0.51	0.87 (0.44–1.7)	0.67
**Stages (I/II or III)**	2.9 (1.67–5.2)	1.9×10^−4^	4.0 (2.02–8.2)	9.0×10^−5^
**Oncotype DX (high/int or low)**	2.38 (1.32–4.27)	0.003		
**MDA114 (high or low)**			2.26 (1.25–4.1)	0.007

We also assessed 5-year DFS rates predicted by the five genomic predictors by calculating area under curves determined by receiver operating characteristic analysis. Only MDA114 and OncoDX showed consistent and significant predictive accuracy in both cohorts (**Figure S2**). Taken together, our findings suggest that these two predictors retain prognostic relevance even after the classic clinicopathological prognostic features are taken into account.

### Genomics predictors in relation to AJCC staging

**Figure 4:**
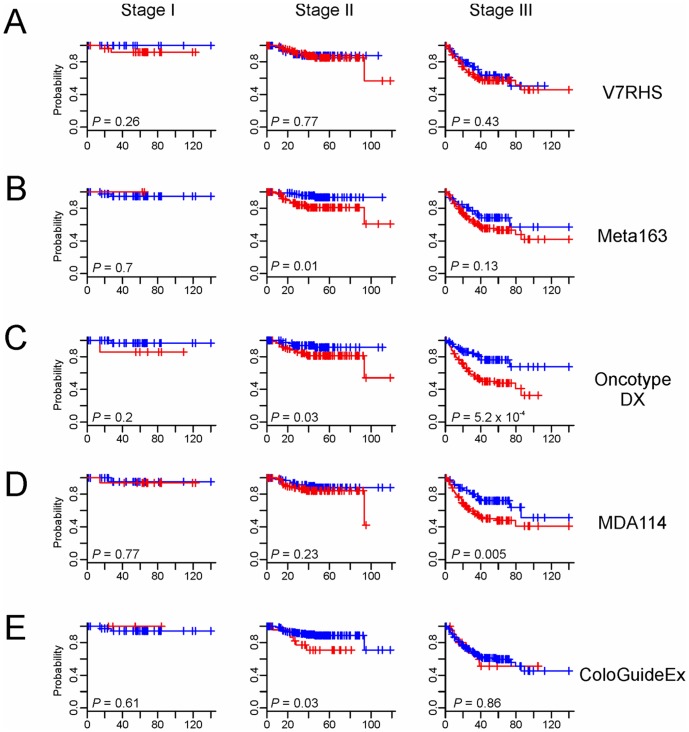
Kaplan–Meier Plots of DFS rates of all patients grouped by AJCC stage. Patients were stratified by risk level according to the five predictors (A to E). *P* values are based on the log-rank test.

We next pooled data from the two cohorts to test the degree to which the predictors are independent from AJCC staging. Meta163, OncoDX, and ColoGuideEx successfully identified high-risk patients with AJCC stage II cancer (**Figure 4B, 4C, 4E**). Likewise, OncoDX and MDA114 showed significantly better prognostication for patients with stage III disease (**Figure 4C and 4D**). These results suggest that some of the genomic predictors may have stage-specific prognostication characteristics. This needs to be validated with a larger, prospective cohort.

### Genomic predictors in relation to adjuvant chemotherapy

Since adjuvant chemotherapy data were available for patients in the AUS cohort, we next sought to determine the association between outcome predicted by of the genomic predictors and adjuvant chemotherapy. We carried out a subset analysis for patients in AJCC stage III (n = 91), a stage for which the benefit of adjuvant chemotherapy has been well established [27]–[29]. Patients with stage III disease were subdivided into two risk groups according to each predictor, and the difference in DFS between the groups was independently assessed.

**Figure 5:**
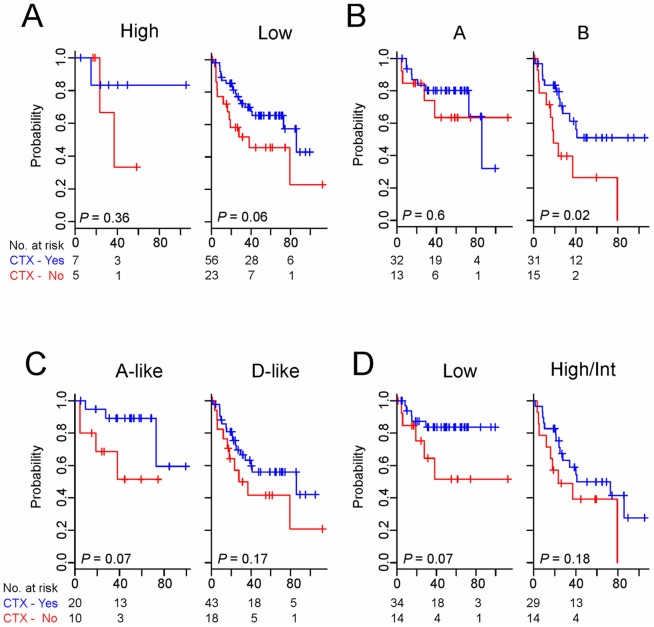
Kaplan-Meier plots of the DFS rates of AUS patients with stage III colorectal cancer in AUS cohort. Patients were stratified by risk level according to the genomic predictors, ColoGuideEx (A), MDA114 (B), Meta163 (C), and OncoDX (D) and grouped by whether they had received adjuvant chemotherapy (CTX) or not. Int, intermediate.

Except for MDA114 predictor, most of genomic predictors failed to show any significant association with adjuvant chemotherapy (**Figure 5**). Subgroup B of MDA114 predictor was only group benefiting significantly from adjuvant chemotherapy (5-year DFS rate, 51% with chemotherapy versus 26% without chemotherapy; *P* = 0.02 by log-rank test, **Figure 5B**). In agreement with the Kaplan-Meier plot and log-rank test, the estimated HR for relapse with adjuvant chemotherapy in subgroup B was 0.31 (95% CI, 0.14–0.73; *P* = 0.007), while HR in subgroup A was 0.67 (95% CI, 0.19–2.34; *P* = 0.5). However, the interaction between MDA114-based subgrouping and adjuvant chemotherapy did not reach significance (*P* = 0.36), suggesting that this association needs to be further tested with a larger prospective cohort.

## Discussion

The objective of this study was to conduct an unbiased comparison of five prognostic genomic predictors and to determine whether their predictions for individual patients were concordant. Of the five predictors, only OncoDX and MDA114 identified, in both tested cohorts, patients with poor prognosis. Although these predictors share only one gene, they predicted similar outcomes, as evidenced by modest but significant correlation for pairwise comparisons. The considerable concordance of outcomes predicted by OncoDX and MDA114 suggests that the gene expression signatures of the two predictors share similar molecular characteristics that are reflected not in an individual gene but in a network of genes.

In multivariate Cox proportional-hazards analysis, both OncoDX and MDA114 were independent variables for predicting DFS in the two cohorts, suggesting that the use of genomic predictors may significantly improve current patient prognostication if the predictors are validated for multiple independent cohorts. Moreover, the two genomic predictors may overcome the limitation of current colorectal cancer staging systems, which do not provide guidance for targeted therapies. Because the gene expression signatures reflect the biological characteristics of each risk group, stratification by genomic predictors would offer new opportunities for rationalized clinical trials to identify subsets of patients who would receive the maximum benefit of a particular targeted treatment.

The discriminatory power of ColoGuideEx for identifying high-risk patients was limited to AJCC stage II in the AUS cohort. Although our result is in good agreement with those of a previous study demonstrating that ColoGuideEx was specific to only patients with stage II disease [11], its stage II-specific prognostification was only marginal in our study, indicating that further validation of this predictor in a larger, independent cohort is necessary. Meta163 and OncoDX also showed stage II-specific prognostication. This finding is consistent with that of a previous study demonstrated the significant accuracy of OncoDX in predicting recurrence in patients with AJCC stage II disease [30].

Because our current cohorts were not trial cohorts, our analyses are not intended to be an exhaustive validation of genomic predictors. For example, all of the patients in the original study for V7RHS were treated only surgically, whereas patients in our study received mixed treatments. Thus, a lack of prognostic ability in our study does not necessarily indicate that one predictor is better or worse than others.

In conclusion, this study has demonstrated that the independently generated genomic predictors MDA114 and OncoDX, with virtually no overlap between their genes, are concordant in predicting outcomes for patients with colorectal cancer. The reason for the lack of overlapping genes among predictors is not completely known; it may due to differences in technological platforms, patient cohorts, or mathematical methods of analysis upon which these assays are based. While genomic predictors have already provided important insights into the biologic heterogeneity of colorectal cancer, the optimal incorporation of these genomic tools into clinical practice remains to be accomplished. These predictors need to be prospectively validated to prove their superiority in predicting risk of recurrence and benefit beyond the use of standard clinicopathological prognosis.

## Supporting Information

Figure S1 **Patients prognosis predicted by five genomic predictors.** A. AUS cohort B. VI cohort
(TIF)

Figure S2 **Prognostic accuracy of the five genome predictors for the AUS (A) and VI (B) cohorts estimated on the basis of areas under the curve (AUC) from the receiver operator characteristic analysis of 5-year DFS.** (A) AUS cohort, (B) VI cohort. ColoEx, ColoGuideEx; CI, 95% confident internal of AUC.
(TIF)

Table S1 **Prognostic signatures in colorectal cancer.**
(DOCX)

Table S2 **Overlap of genes among prognostic signatures.**
(DOCX)

Table S3 **Cross-comparison of membership of patients in AUS cohort according to five predictors.**
(DOCX)

Table S4 **Concordance of the five genomic predictors in grouping VI patients by risk level.**
(DOCX)

Table S5 **Univariate Cox proportional hazard regression analyses of DFS with clinical variables and genomic predictors in AUS cohort.**
(DOCX)

Table S6 **Univariate Cox proportional hazard regression analyses of OS with clinical variables and genomic predictors in VI cohort.**
(DOCX)
