# Correction: Comparison of Prognostic Genomic Predictors in Colorectal Cancer

**DOI:** 10.1371/annotation/d5ab6ce1-8f47-4419-8642-c0b010699ef0

**Published:** 2013-12-17

**Authors:** Yun-Yong Park, Sung Sook Lee, Jae Yun Lim, Sang Cheol Kim, Sang Bae Kim, Bo Hwa Sohn, In-Sun Chu, Sang Cheul Oh, Eun Sung Park, Woojin Jeong, Sung Soo Kim, Scott Kopetz, Ju-Seog Lee

Affiliation number 9 for the 11th and last authors is incorrect. The correct affiliation is: Department of Biochemistry and Molecular Biology, Medical Research Center for Bioreaction to Reactive Oxygen Species and Biomedical Science Institute, School of Medicine, Kyunghee University, Seoul, Korea. 

