# Supplementary figures and images for: Comparison of Prognostic Genomic Predictors in Colorectal Cancer
Source: PLoS One. 2013 Apr 23;8(4):e60778. doi: 10.1371/journal.pone.0060778 (PMC3634034; doi:10.1371/journal.pone.0060778)

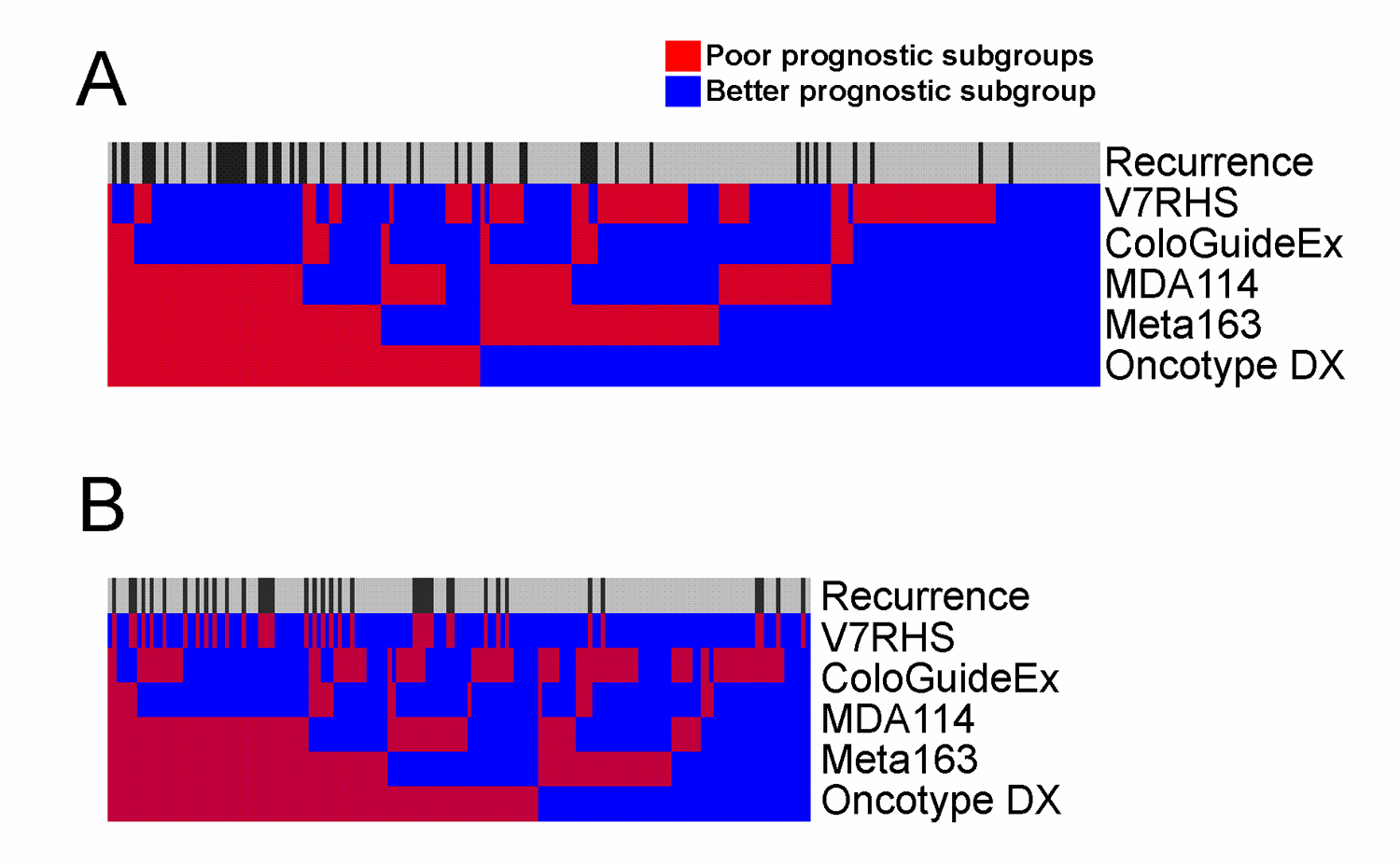

Supplement: Figure S1 — Patients prognosis predicted by five genomic predictors. A. AUS cohort B. VI cohort (TIF) [file pone.0060778.s001.tif]

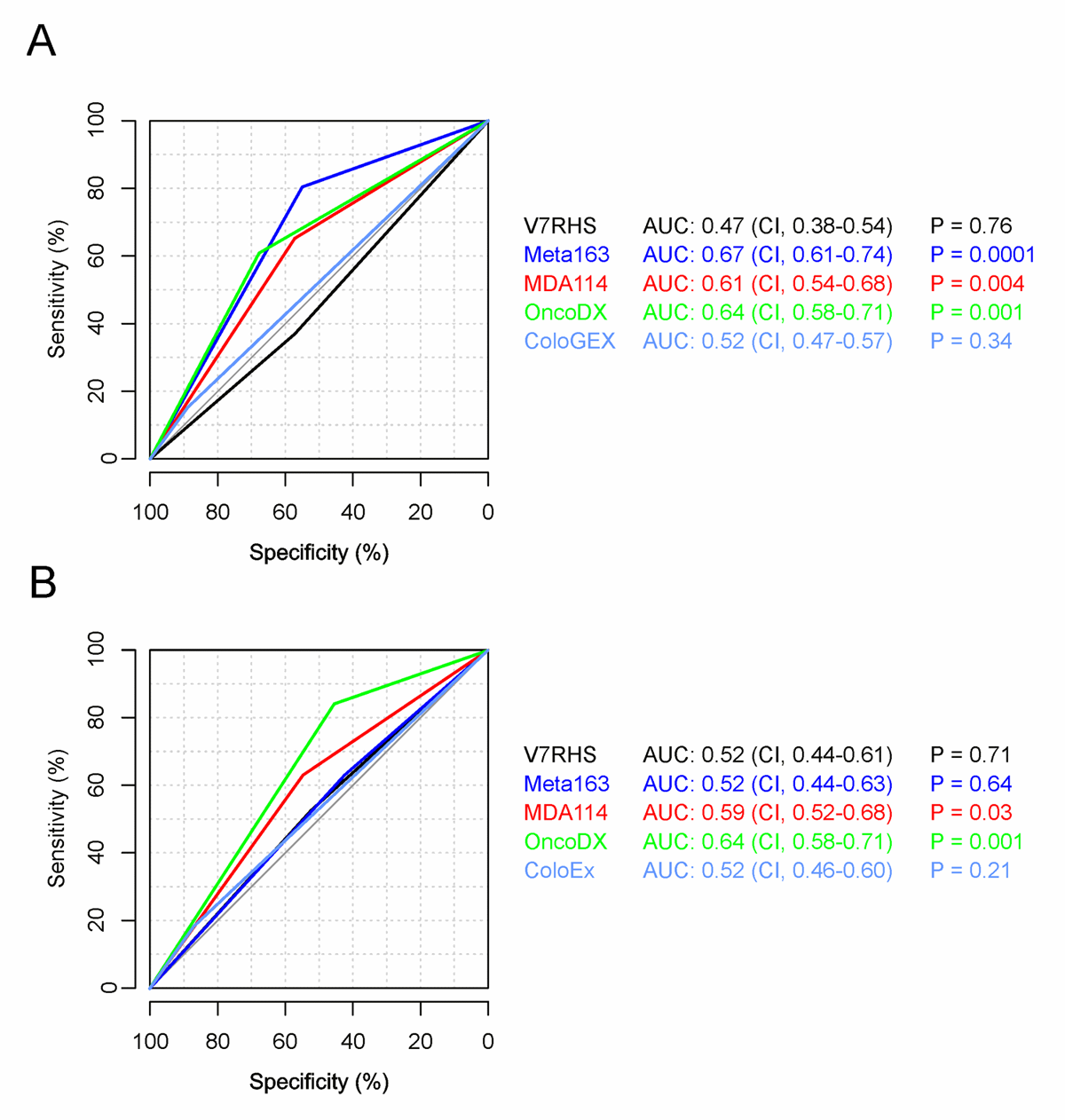

Supplement: Figure S2 — Prognostic accuracy of the five genome predictors for the AUS (A) and VI (B) cohorts estimated on the basis of areas under the curve (AUC) from the receiver operator characteristic analysis of 5-year DFS. (A) AUS cohort, (B) VI cohort. ColoEx, ColoGuideEx; CI, 95% confident internal of AUC. (TIF) [file pone.0060778.s002.tif]
