# Supplementary material for: Comparison of Prognostic Genomic Predictors in Colorectal Cancer
Source: PLoS One. 2013 Apr 23;8(4):e60778. doi: 10.1371/journal.pone.0060778 (PMC3634034; doi:10.1371/journal.pone.0060778)
Supplement: Table S1 — Prognostic signatures in colorectal cancer. (DOCX) [file pone.0060778.s003.docx]

| **Signatures** | **Genes (Probes)** | **Reference** |
| --- | --- | --- |
| Veridex 7-gene relapse hazard score (V7RHS) | 7 (7) | 7 |
| Metastasis-associated 163 genes (Meta163) | 121 (163) | 5 |
| Oncotype DX Colon Cancer Assay (OncoDX RS) | 7 (7) | 8 |
| MDACC 114-gene predictor (MDA114) | 86 (114) | 6 |
| ColoGuideEx | 13 (13) | 11 |

**Table S1.** Prognostic signatures in colorectal cancer.
