# Supplementary material for: Comparison of Prognostic Genomic Predictors in Colorectal Cancer
Source: PLoS One. 2013 Apr 23;8(4):e60778. doi: 10.1371/journal.pone.0060778 (PMC3634034; doi:10.1371/journal.pone.0060778)
Supplement: Table S2 — Overlap of genes among prognostic signatures. (DOCX) [file pone.0060778.s004.docx]

|  | V7RHS | ColoGuideEx | Meta163 | OncoDX | MDA114 |  | unique genes | Total Probes |
| --- | --- | --- | --- | --- | --- | --- | --- | --- |
| V7RHS |  | 0 | 0 | 0 | 0 |  | 7 | 7 |
| ColoGuideEx | 0 |  | 2 | 0 | 1 |  | 13 | 13 |
| Meta163 | 0 | 2 |  | 1 | 7 |  | 121 | 163 |
| OncoDX | 0 | 0 | 1 |  | 1 |  | 7 | 7 |
| MDA114 | 0 | 1 | 7 | 1 |  |  | 86 | 114 |

**Table S2.** Overlap of genes among prognostic signatures.
