# Supplementary material for: Comparison of Prognostic Genomic Predictors in Colorectal Cancer
Source: PLoS One. 2013 Apr 23;8(4):e60778. doi: 10.1371/journal.pone.0060778 (PMC3634034; doi:10.1371/journal.pone.0060778)
Supplement: Table S3 — Cross-comparison of membership of patients in AUS cohort according to five predictors. (DOCX) [file pone.0060778.s005.docx]

|  | **Classification** | **Total Patients** |  | **ColoGuideEx** | |  | **Meta163** | |  | **Oncotype DX** | |  | **MDA114** | |
| --- | --- | --- | --- | --- | --- | --- | --- | --- | --- | --- | --- | --- | --- | --- |
| **Classification** |  |  |  | **High** | **Low** |  | **D-like** | **A-like** |  | **High/Int** | **Low** |  | **High** | **Low** |
| **Total Patients** |  |  |  | 27 (7) | 202 (43) |  | 118 (40) | 111 (10) |  | 86 (32) | 143 (18) |  | 107 (32) | 122 (18) |
|  |  |  |  |  |  |  |  |  |  |  |  |  |  |  |
| **V7RHS** | **High** | 96 (18) |  | 13 (3) | 83 (15) |  | 45 (13) | 51 (5) |  | 18 (5) | 78 (13) |  | 22 (5) | 74 (13) |
|  | **Low** | 133 (32) |  | 14 (4) | 119 (28) |  | 73 (27) | 60 (5) |  | 68 (27) | 65 (5) |  | 85 (27) | 48 (5) |
|  |  |  |  |  |  |  |  |  |  |  |  |  |  |  |
| **ColoGuideEx** | **High** | 27 (7) |  |  |  |  | 20 (7) | 7 (0) |  | 14 (4) | 13 (3) |  | 10 (3) | 17 (4) |
|  | **Low** | 202 (43) |  |  |  |  | 98 (33) | 104 (10) |  | 72 (28) | 130 (15) |  | 97 (29) | 105 (14) |
|  |  |  |  |  |  |  |  |  |  |  |  |  |  |  |
| **Meta163** | **D-like** | 118 (40) |  |  |  |  |  |  |  | 63 (28) | 55 (12) |  | 66 (27) | 52 (13) |
|  | **A-like** | 111 (10) |  |  |  |  |  |  |  | 2 (4) | 88 (6) |  | 41 (5) | 70 (5) |
| **Oncotype DX** | **High/Int** | 86 (32) |  |  |  |  |  |  |  |  |  |  | 60 (26) | 26 (6) |
|  | **Low** | 143 (18) |  |  |  |  |  |  |  |  |  |  | 47 (6) | 96 (12) |

**Table S3** Cross-comparison of membership of patients in AUS cohort according to five predictors

Numbers in parenthesis represent recurrence
