# Supplementary material for: Comparison of Prognostic Genomic Predictors in Colorectal Cancer
Source: PLoS One. 2013 Apr 23;8(4):e60778. doi: 10.1371/journal.pone.0060778 (PMC3634034; doi:10.1371/journal.pone.0060778)
Supplement: Table S4 — Concordance of the five genomic predictors in grouping VI patients by risk level. (DOCX) [file pone.0060778.s006.docx]

**Table S4.** Concordance of the five genomic predictors in grouping VI patients by risk level.

|  | **V7RHS** | **Meta163** | **Oncotype DX** | **MDA114** | **ColoGuideEx** |
| --- | --- | --- | --- | --- | --- |
| **V7RHS** | 1 | 0.16 | 0.26 | 0.27 | 0.02 |
| **Meta163** | 0.16 | 1 | 0.16 | 0.19 | 0.13 |
| **Oncotype DX** | 0.26 | 0.16 | 1 | 0.39 | 0.04 |
| **MDA114** | 0.27 | 0.19 | 0.39 | 1 | 0.06 |
| **ColoGuideEx** | 0.02 | 0.13 | 0.04 | 0.06 | 1 |

Correlation was quantified using Cramer’s V statistics
