# Supplementary material for: Comparison of Prognostic Genomic Predictors in Colorectal Cancer
Source: PLoS One. 2013 Apr 23;8(4):e60778. doi: 10.1371/journal.pone.0060778 (PMC3634034; doi:10.1371/journal.pone.0060778)
Supplement: Table S5 — Univariate Cox proportional hazard regression analyses of DFS with clinical variables and genomic predictors in AUS cohort. (DOCX) [file pone.0060778.s007.docx]

**Table S5** Univariate Cox proportional hazard regression analyses of DFS with clinical variables and genomic predictors in AUS cohort

|  | **Hazard Ratio (95% CI)** | ***P*-value** |
| --- | --- | --- |
| **Sex (male or female)** | 1.01 (0.62 – 1.92) | 0.74 |
| **Age (>70 or not)** | 0.75 (0.41 - 1.34) | 0.33 |
| **AJCC stages (I/II, or III)** | 3.6 (2.0 - 6.7) | 1.7 x 10^-5^ |
| **Location (colon or rectum)** | 1.0 (0.45 - 2.2) | 0.98 |
| **Chemotherapy (yes or no)** | 1.9 (1.01 – 3.3) | 0.02 |
| **V7RHS (high or low)** | 0.84 (0.47 – 1.51) | 0.57 |
| **ColoGuideEx (high or low)** | 1.42 (0.64 - 3.16) | 0.39 |
| **Meta163 (D-like or A-like)** | 4.5 (2.2 - 9.0) | 2.1 x 10^-5^ |
| **Oncotype DX (iigh/int or low)** | 2.9 (1.6 - 5.3) | 2.2 x 10^-4^ |
| **MDA114 (high or low)** | 2.2 (1.2 - 3.9) | 0.008 |
