# Supplementary material for: Comparison of Prognostic Genomic Predictors in Colorectal Cancer
Source: PLoS One. 2013 Apr 23;8(4):e60778. doi: 10.1371/journal.pone.0060778 (PMC3634034; doi:10.1371/journal.pone.0060778)
Supplement: Table S6 — Univariate Cox proportional hazard regression analyses of OS with clinical variables and genomic predictors in VI cohort. (DOCX) [file pone.0060778.s008.docx]

**Table S6** Univariate Cox proportional hazard regression analyses of OS with clinical variables and genomic predictors in VI cohort

|  | **Hazard Ratio (95% CI)** | ***P*-value** |
| --- | --- | --- |
| **Sex (male or female)** | 1.0 (0.56 – 2.0) | 0.8 |
| **Age (>70 or not)** | 0.64 (0.32 - 1.28) | 0.2 |
| **AJCC stages (I/II, or III)** | 5.3 (2.4 - 11.6) | 2.7 x 10^-5^ |
| **V7RHS (high or low)** | 0.86 (0.45 – 1.6) | 0.65 |
| **ColoGuideEx (high or low)** | 1.4 (0.58 - 3.0) | 0.48 |
| **Meta163 (D-like or A-like)** | 1.3 (0.67 - 2.53) | 0.42 |
| **Oncotype DX (high/int or low)** | 3.9 (1.6 - 9.4) | 0.002 |
| **MDA114 (high or low)** | 2.1 (1.1 - 4.0) | 0.03 |
